# Exploration of the Proteomic Landscape of Small Extracellular Vesicles in Serum as Biomarkers for Early Detection of Colorectal Neoplasia

**DOI:** 10.3389/fonc.2021.732743

**Published:** 2021-09-13

**Authors:** Li-Chun Chang, Yi-Chiung Hsu, Han-Mo Chiu, Koji Ueda, Ming-Shiang Wu, Chiun-How Kao, Tang-Long Shen

**Affiliations:** ^1^Department of Internal Medicine, National Taiwan University Hospital, Taipei, Taiwan; ^2^Health Management Center, National Taiwan University Hospital, Taipei, Taiwan; ^3^Department of Biomedical Science and Engineering, National Central University, Taoyuan, Taiwan; ^4^Cancer Precision Medicine Center, Japanese Foundation of Cancer Research, Tokyo, Japan; ^5^Department of Statistics, Tamkang University, New Taipei City, Taiwan; ^6^Department of Plant Pathology and Microbiology, National Taiwan University, Taipei, Taiwan; ^7^Center for Biotechnology, National Taiwan University, Taipei, Taiwan; ^8^Genome and Systems Biology Degree Program, National Taiwan University, Taipei, Taiwan

**Keywords:** colorectal cancer, blood test, small extracellular vesicle, proteome, biomarker

## Abstract

**Background:**

Patient participation in colorectal cancer (CRC) screening *via* a stool test and colonoscopy is suboptimal, but participation can be improved by the development of a blood test. However, the suboptimal detection abilities of blood tests for advanced neoplasia, including advanced adenoma (AA) and CRC, limit their application. We aimed to investigate the proteomic landscape of small extracellular vesicles (sEVs) from the serum of patients with colorectal neoplasia and identify specific sEV proteins that could serve as biomarkers for early diagnosis.

**Materials and Methods:**

We enrolled 100 patients including 13 healthy subjects, 12 non-AAs, 13 AAs, and 16 stage-I, 15 stage-II, 16 stage-III, and 15 stage-IV CRCs. These patients were classified as normal control, early neoplasia, and advanced neoplasia. The sEV proteome was explored by liquid chromatography-tandem mass spectrometry. Generalized association plots were used to integrate the clustering methods, visualize the data matrix, and analyze the relationship. The specific sEV biomarkers were identified by a decision tree *via* Orange3 software. Functional enrichment analysis was conducted by using the Ingenuity Pathway Analysis platform.

**Results:**

The sEV protein matrix was identified from the serum of 100 patients and contained 3353 proteins, of which 1921 proteins from 98 patients were finally analyzed. Compared with the normal control, subjects with early and advanced neoplasia exhibited a distinct proteomic distribution in the data matrix plot. Six sEV proteins were identified, namely, GCLM, KEL, APOF, CFB, PDE5A, and ATIC, which properly distinguished normal control, early neoplasia, and advanced neoplasia patients from each other. Functional enrichment analysis revealed that APOF^+^ and CFB^+^ sEV associated with clathrin-mediated endocytosis signaling and the complement system, which have critical implications for CRC carcinogenesis.

**Conclusion:**

Patients with colorectal neoplasia had a distinct sEV proteome expression pattern in serum compared with those patients who were healthy and did not have neoplasms. Moreover, the six identified specific sEV proteins had the potential to discriminate colorectal neoplasia between early-stage and advanced neoplasia. Collectively, our study provided a six-sEV protein biomarker panel for CRC diagnosis at early or advanced stages. Furthermore, the implication of the sEV proteome in CRC carcinogenesis *via* specific signaling pathways was explored.

## Introduction

Colorectal cancer (CRC) ranks as the third most common cancer and the second leading cause of cancer-related deaths worldwide; therefore, it is a critical public health issue (1). In Taiwan, CRC incidence was 42.9 cases per 100,000 residents in 2017 and higher than lung cancer, breast cancer, and hepatoma. Early detection and robust screening for CRC could significantly reduce mortality from CRC (2). Considerable efforts have substantially improved CRC screening participation, which has resulted in a 3.3% decrease per year in CRC incidence rates from 2011 to 2016 (3). The most commonly used modalities for screening CRC are stool-based tests and colonoscopy. Among the various screening modalities, colonoscopy offers confirmatory evidence of disease and reduces mortality rates by 67% (4, 5). However, the inconvenience, invasiveness, and cost of colonoscopy limit patient compliance to screening. Thus, a population-based randomized control trial demonstrated that fecal immunochemical tests (FITs) detected as many CRCs as colonoscopy as a first-line screening modality (6). Currently, stool-based FIT and DNA tests are available for screening CRC. Although FIT profoundly contributes to reducing CRC mortality, low sensitivity to precancerous lesions has hampered its clinical utility (7–9). Although stool DNA tests show a better detection for precancerous lesions and early cancers than FIT, participation in stool-based screening remains suboptimal because of the negative acceptance of fecal specimen collection, handling, and transportation to the clinic (10, 11). A blood test could provide an additional 6.5% participation than FIT for screening (12). Not surprisingly, there is a preference for blood-based screening to improve the limited acceptance of either colonoscopy or stool-based tests, highlighting the need and potential merit for developing highly robust blood-based tests for identifying colorectal neoplasia.

Small extracellular vesicles (sEVs) are membrane-bound vesicles ranging from 30 to 150 nm in diameter that are secreted by various cell types. They encapsulate multiple proteins, nucleic acids, and other biomolecules with either the physiological or pathological status of parental cells (13). sEVs transport biomolecules between cells and play an essential role in intercellular communication locally and systematically. Moreover, they contribute to cancer formation and progression by facilitating tumor angiogenesis and metastasis. The contents of sEVs are protected from external proteases and other enzymes and remain biologically stable. sEVs isolated from breast cancer demonstrated high levels of stable phosphorylated proteins and provided a window into the molecular mechanism underlying cancer progression and metastasis (14). Thus, sEVs are helpful biomarkers for the early detection of cancer. Previous studies have explored Glypican-1-containing sEVs that may play a role in diagnosing pancreatic cancer (15) and CRC (16). Moreover, CD147-containing sEVs in plasma from CRC patients are also higher than those from healthy subjects (17).

Proteins, not only genes composed of nucleic acids, are responsible for the phenotype of cancer cells. Therefore, it is crucial to elucidate the mechanisms of disease by investigating the proteome. Proteomics is a large-scale investigation of proteins that can comprehensively map the biological processes of carcinogenesis (18). The proteome is more complex than the genome because of alternative transcription initiation, alternative splicing, RNA editing, proteolytic processing, and posttranslational modifications. Proteins in the blood have the potential to serve as biomarkers for detecting CRC. However, the detection of low abundance proteins in the blood remains challenging (10). sEVs encapsulate proteins or present proteins on the surface, which provides a window for amplifying the low abundance proteins in the blood through the purification and enrichment of sEVs. As mentioned above, growing evidence supports that proteins contained within sEVs could be used to detect various cancers, including CRC (14–16).

In this study, we explored the proteomic landscape of sEVs specific to advanced adenoma (AA) and CRC and their potential as biomarkers for detecting colorectal neoplasia.

## Methods

### Patients

Patients who underwent colonoscopy at National Taiwan University Hospital for screening, surveillance, or symptoms and received endoscopic treatment or surgery were included prospectively. The patients were categorized based on colonoscopic findings and histologic diagnosis. Blood samples were collected chronologically within 6 years (from 2014 to 2019). Participants who had a history of inflammatory bowel disease or hereditary CRC, such as Lynch syndrome, familial adenomatous polyposis, or hyperplastic polyposis, were excluded. Participants with active malignancy within 5 years before the diagnosis of CRC were also excluded. This study protocol was approved by the Institutional Review Board of the National Taiwan University Hospital (No. 201912055RINB). All data used in this study were anonymous without any identifiable personal information.

### Definition

Colorectal neoplasms were classified as non-AA, AA, and CRC according to the 2019 WHO classification system for tumors of the digestive system (19). AA was defined as a lesion 10 mm or larger in diameter or with a villous component or high-grade dysplasia. Healthy controls were defined as subjects with a negative colonoscopic finding. Each subcategory was defined as follows: normal control including healthy control and non-AA; early neoplasia including AA and stage-I CRC; advanced neoplasia including stage II–IV CRC. The cecum to splenic flexure was defined as the proximal colon, and the descending colon to the rectum was defined as the distal colon.

### Blood Collection and Isolation of sEVs From Human Serum

Whole blood was collected in a BD Vacutainer™ SST™ II Advanced tube and processed within 2 h after blood was drawn. We centrifuged the blood SST tubes for 10 min at 3000 g in a prechilled centrifuge set at 25°C, and we used a swing-out rotor with appropriate buckets. We carefully pipetted off the serum supernatant, the serum was aliquoted and kept at -80°C until use, and freeze-thawing cycles were avoided as much as possible after frozen storage. sEVs were purified from human serum samples using size exclusion chromatography on a drip with extracellular vesicle isolation by size exclusion chromatography on an EVSecond L70^®^ drip column (GL Sciences, Tokyo, Japan). The column was initially equilibrated with 700 μl of fetal bovine serum twice, followed by three washing steps using 1500 μl of PBS. After washing, 100 μl of the collected human serum sample was loaded onto the column, followed by the collection of 12 consecutive fractions in 100 μl of PBS. CD9 expression in these fractions was analyzed using Western blotting, and CD9-positive fractions were recognized as the sEV-rich portion. The sEVs obtained using this system were previously authenticated with transmission electron microscopy and Western blotting. The diameters of the particles in the sEV fractions were analyzed with a NanoSight™ LM10.

### Liquid Chromatography-Tandem Mass Spectrometry (LC-MS) Analysis

The sEV-containing eluates of EVSecond L70 columns (GL Sciences, Tokyo, Japan) were dried and resolved in 20 mM HEPES-NaOH (pH 8.0), 12 mM sodium deoxycholate, and 12 mM sodium N-lauroyl sarcosinate. In the elution step, we collected eluate fractions continuously (100 µl/fraction). Then, the 4th to 6th fractions were combined (300 µl) and dried by vacuum evaporation. Following reduction with 20 mM dithiothreitol at 100°C for 10 min and alkylation with 50 mM iodoacetamide at ambient temperature for 45 min, proteins were digested with 5 μl of immobilized trypsin (Thermo Fisher Scientific, Inc., Waltham, MA, USA) on a plate shaker (TAITEC, M-BR-024) at 1000 rpm at 37°C for 6 h to mix the trypsin beads. After removing sodium deoxycholate and sodium N-lauroyl sarcosinate by ethyl acetate extraction, the resulting peptides were desalted by an Oasis HLB μElution plate (Waters Corp., Milford, MA, USA) and subjected to mass spectrometric analysis. The Oasis plate was prewetted with 500 µl 70% acetonitrile and equilibrated with 500 µl 0.1% TFA in 2% acetonitrile at 250 µl/min. Following sample loading at 100 µl/min, plates were washed twice with 500 µl 0.1% TFA in 2% acetonitrile at 250 µl/min. Peptides were eluted with 100 μl 70% acetonitrile at 100 μl/min and subsequently dried by vacuum evaporation. Peptides were analyzed using an LTQ-Orbitrap-Velos mass spectrometer (Thermo Fisher Scientific, Inc.) combined with an UltiMateTM 3000 RSLCnano-flow HPLC system (Thermo Fisher Scientific, Inc.). Using 0.1% formic acid as Solvent A and 0.1% formic acid in acetonitrile as Solvent B, peptides were separated on a C18 Chip-column (75 µm × 200 mm, Nikkyo Technos, Tokyo, Japan) by a gradient of Solvent B 2–30% for 95 min and 30–95% for 15 min at a flow rate of 250 nl/min. The eluted peptides were ionized with a spray voltage of 2000 V, and MS data were acquired in a data-dependent fragmentation method in which the survey scan was acquired between m/z 400 and 1600 at a resolution of 60,000 with an automatic gain control (AGC) target value of 1.0 × 106 ion counts. The top 20 intense precursor ions in each survey scan were subjected to low-resolution MS/MS acquisitions using normal CID scan mode with an AGC target value of 5,000 ion counts in the linear ion trap. Protein identification and quantification were performed using MaxQuant software (https://www.biochem.mpg.de/5111795/maxquant). The MS/MS spectra were searched against the Homo sapiens protein sequence database in SwissProt using Proteome Discoverer 2.4 software (Thermo Scientific), in which peptide identification filters were set at a false discovery rate <1%. The minimum peptide length was 6. The single peptide identification was used. Only “Razor + unique peptides” were used for the calculation of relative protein concentration. Candidate proteins were selected using the following criteria: commonly expressed in sEVs from all of the subjects with colorectal neoplasia; plasma membrane protein expressed on the sEV membrane; and higher expression level in patients with colorectal neoplasia than in healthy individuals.

### Identification and Label-Free Quantification of Proteins

Protein identification and label-free quantification were performed using Proteome Discoverer 2.2 software (Thermo Fisher Scientific). For protein identification, the LC-MS dataset was searched against the SwissProt Human Database with the Mascot (Matrix Science) or Sequest HT (Thermo Fisher Scientific) database search engine, where FDR <1% was set as the peptide identification threshold. For label-free quantification and data normalization, the Minora Feature Detector node in the Processing workflow and the Feature Mapper node followed by the Precursor Ions Quantifier node in the Consensus workflow were used with default parameters in Proteome Discoverer 2.2 software.

### Cluster Analysis

A generalized association plot (GAP, http://gap.stat.sinica.edu.tw/Software/GAP/index.htm) was used to visualize the expression patterns and identify the outliers in patient proteins (20, 21). We chose the proximity measure of rows with Euclidean distance and Pearson correlation for the column to create the data matrix and two proximity matrices. After deciding on the parameters, GAP used matrix visualization (MV) to present three matrix data. Because the data matrix was standardized, a symmetric blue (negative) to white (zero) to red (positive) color spectrum (Display Condition set to Center: Matrix) was used to represent the range (−9.78, 9.78). In this study, three MV maps were sorted by HCT–R2E dendrograms (22) for 1921 protein expression on 98 patient’s data. The two proximity matrices are shown in **Supplementary Figure S1**.

### Functional Enrichment Analysis

Ingenuity Pathway Analysis (IPA) (QIAGEN company, Redwood City, CA, USA), a web-based computational platform, was used to conduct functional enrichment analysis of genes. A total of 1921 differentially expressed proteins (>2-fold change) were analyzed by the core analysis enrichment tool based on the IPA database. Canonical pathways and upstream regulator analysis found by core analysis in IPA were given a p-value.

### Statistical Analysis

In this study, we constructed a decision tree in Orange3 (https://orangedatamining.com/) to extract six essential proteins. The parameters of the decision tree in Orange3 were set to induce a binary tree which splits parent node into two child nodes, a minimum number of instances in leaves equal to 1, forbids the algorithm to split the nodes with less than 1 (do not split subsets smaller than = 1), and limit the maximal tree depth set to default. The visualization for the result also used the component of Tree Viewer in Orange3.

## Results

### Demographics and Clinical Information of Eligible Subjects

A total of 100 subjects were enrolled in the present study. The clinical demographics are listed in **Table 1**. The mean age was 62.2 years old, and 50% of them were male. The indications for colonoscopy included screening (67%), surveillance (3%), and symptomatic (30%). Thirteen healthy controls, 12 non-AAs, 13 AAs, 16 stage-I CRCs, 15 stage-II CRCs, 16 stage-III CRCs, and 15 stage-IV CRCs were eligible and were included in the study. Among all colorectal neoplasias, 33 (37.5%) lesions were located in the proximal colon, as confirmed by histological diagnosis.

**Table 1:** Demographics and clinical information of the study subjects.

Clinical information	N = 100
Mean age, years (SD)	62.2 (14.4)
Gender	
Male, n (%)	50 (50.0)
Female, n (%)	50 (50.0)
Smoking, n (%)	
Non-smoker	34 (34.0)
Current smoker	3 (3.0)
Ex-smoker	5 (5.0)
Indication for colonoscopy, n (%)	
Screening	67 (67.0)
Surveillance	3 (3.0)
Symptomatic	30 (30.0)
Pathology, n (%)	
Healthy control	13 (13.0)
Non-AA	12 (12.0)
AA	13 (13.0)
Invasive cancer	
Stage I	16 (25.8)
Stage II	15 (24.2)
Stage III	16 (25.8)
Stage IV	15 (24.2)
Proximal lesion, n (%)	33 (37.5)

Non-AA, non-advanced adenoma; AA, advanced adenoma.

### Generalized Association Plots

**Figure 1:**
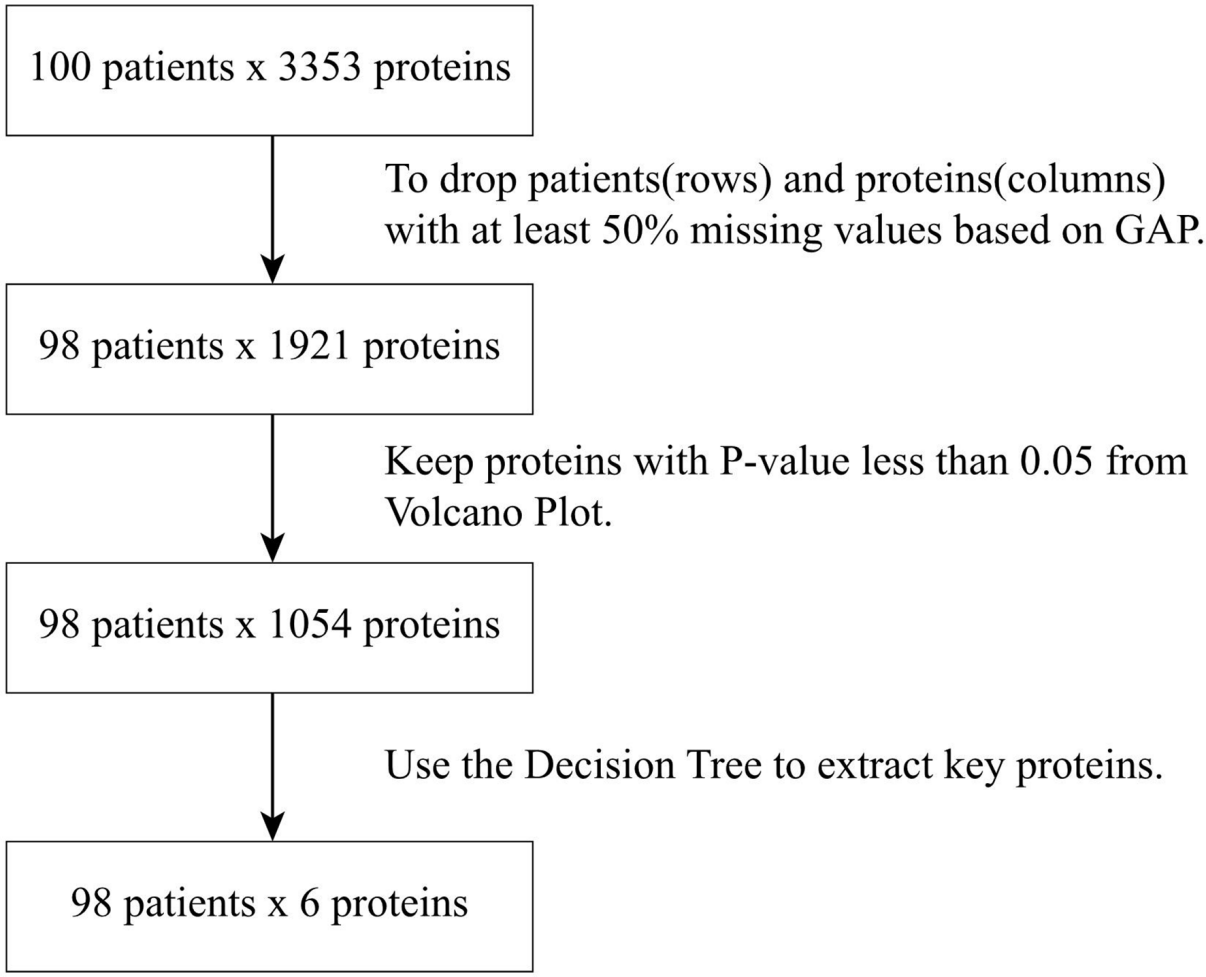
Flowchart of protein selection and data analysis procedures.

**Figure 2:**
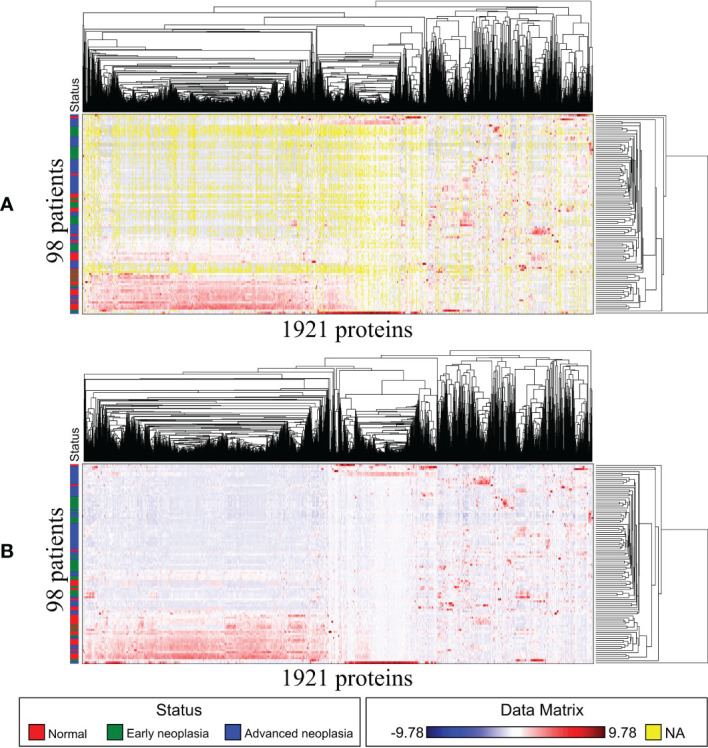
Matrix visualization for exosome proteins also sorted by HCT–R2E seriations: 98 patients by 1921 proteins. The Euclidean distance map for 98 patients and the Pearson correlation map for 1921 proteins are not shown due to space limitations. **(A)** Matrix visualization before imputation. **(B)** Matrix visualization after imputation.

The raw data of the sEV proteome containing 3353 proteins were obtained from the 100 patients. To minimize the influence of missing data on the correlation between proteins and patients, patients (rows) or proteins (columns) with missing data of more than 50% were excluded. Accordingly, a total of 1921 proteins from 98 patients were subjected to analysis as a sEV proteome dataset. The flowchart of protein selection is demonstrated in **Figure 1**. Because the expression difference in each of the 1921 sEV proteins was significant, standardization was used to normalize the protein expression data. Then, we used GAP, which integrated clustering methods and visualization of the data matrix, and two proximity matrices to analyze the relationship between patients and proteins. MV was employed for the demonstration of the sEV protein data matrix to achieve a better understanding of the association between the patients and proteins, and the result is illustrated in **Figure 2A**. Data from the normal subjects were mostly concentrated at the bottom of the data matrix plot, and approximately 40% of the analyzed proteins had prominently high expression. Apparently, data from the patients with early and advanced neoplasia were presented at the top of the data matrix plot; nevertheless, the data was mixed and difficult to distinguish from each other. To minimize the influence of the missing value (yellow), the missing values in the data matrix were imputed with the average expression value of each protein accordingly. The result after imputation is shown in **Figure 2B**. The patients with early and advanced neoplasia continued to display a different expression pattern of the sEV proteins from normal subjects. Based on these GAP results, we were confident in identifying the specific sEV proteins that distinguished patients with colorectal neoplasia from those who were classified as healthy and/or nonneoplastic.

### Volcano Plot

**Figure 3:**
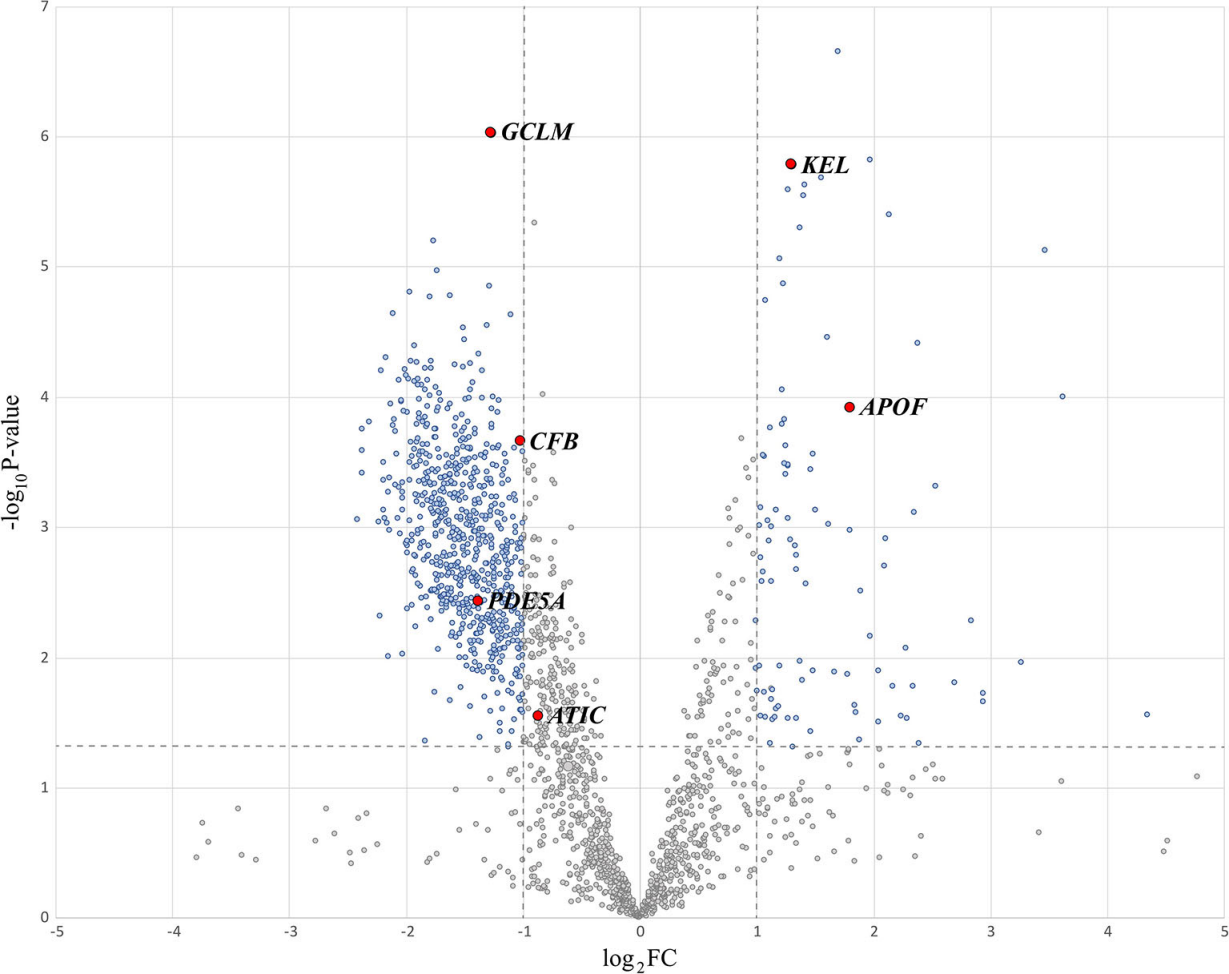
Volcano plot highlighting significant proteins.

Next, we calculated the fold change (FC) and p-value < 0.05 of the 1921 sEV proteins from 98 normal and tumor patients and drew a volcano map, as shown in **Figure 3**. The blue dots in the figure represent proteins with a p-value less than 0.05 and log_2_ (FC) absolute value greater than 1. The proteins whose log_2_ (FC) was positive had higher expression in tumor status. Since we wanted to analyze the proteins with significant differences, 1054 proteins with a p-value less than 0.05 were selected for further examination. Among them, 170 were upregulated, and 884 proteins were downregulated in CRC neoplasia-derived sEVs.

### Identification of EV Proteins Specific to Colorectal Neoplasia

**Figure 4:**
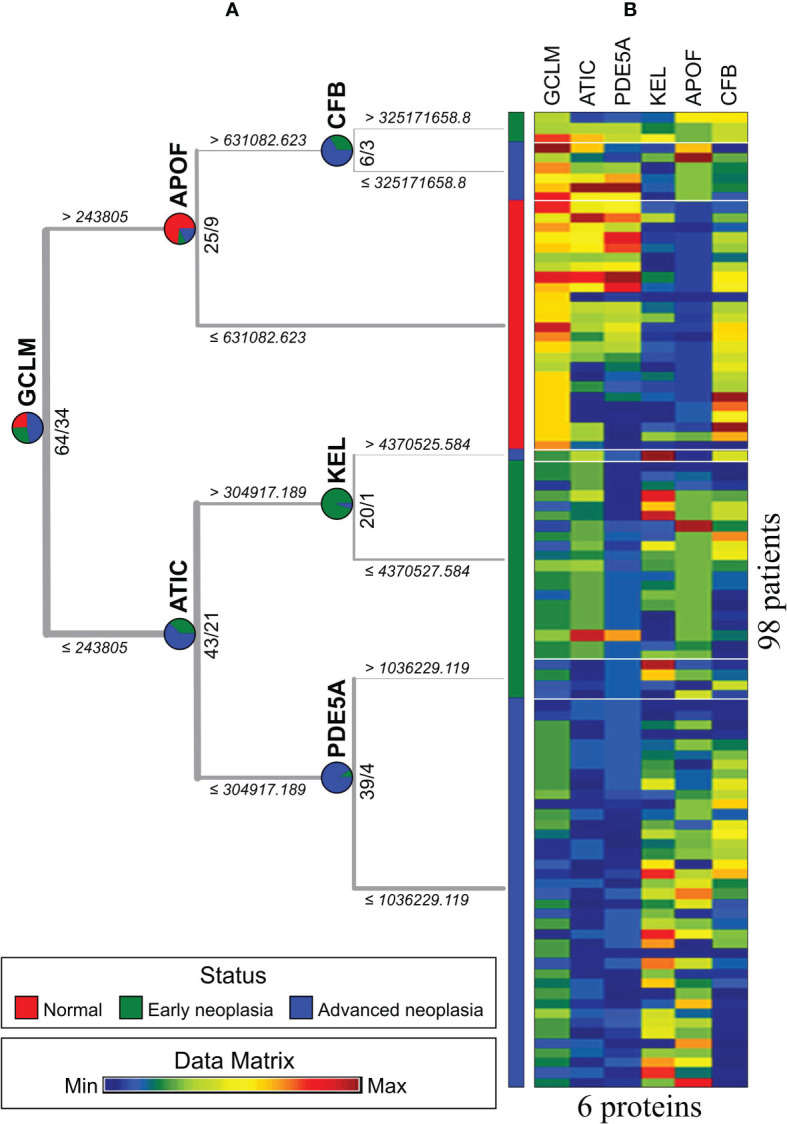
Three status types were classified by an exosome protein data matrix for 98 patients. **(A)** The result of the decision tree. **(B)** Matrix visualization based on decision tree results.

To identify critical proteins capable of distinguishing three subcategories, normal, early neoplasia and advanced neoplasia, we used a decision tree to classify the sEV protein data (98 patients by 1054 proteins). The visualization of the decision tree result is shown in **Figure 4A**. We found that 6 sEV proteins (GCLM, KEL, APOF, CFB, PDE5A, and ATIC) can perfectly distinguish patients from the three different subcategories in a differential expression manner. **Figure 4B** presents the expression values of these 6 sEV proteins from 98 patients by MV. In **Figure 4**, the patients of normal group (red) can be distinguished by GCLM^high^ and APOF^low^, and GCLM^low^, ATIC^low^/GCLM^high^, CFB^low^ can distinguish most patients with early neoplasia (green) and advanced neoplasia (blue), and additional predictors of early or advanced neoplasia are PED5A^low^, KEL^high^ and PED5A^high^, KEL^low^, respectively. Consistently, these six essential sEV proteins indicated by the red dots on the volcano map in **Figure 3** are shown to be statistically significant.

### Functional Enrichment Analysis

**Figure 5:**
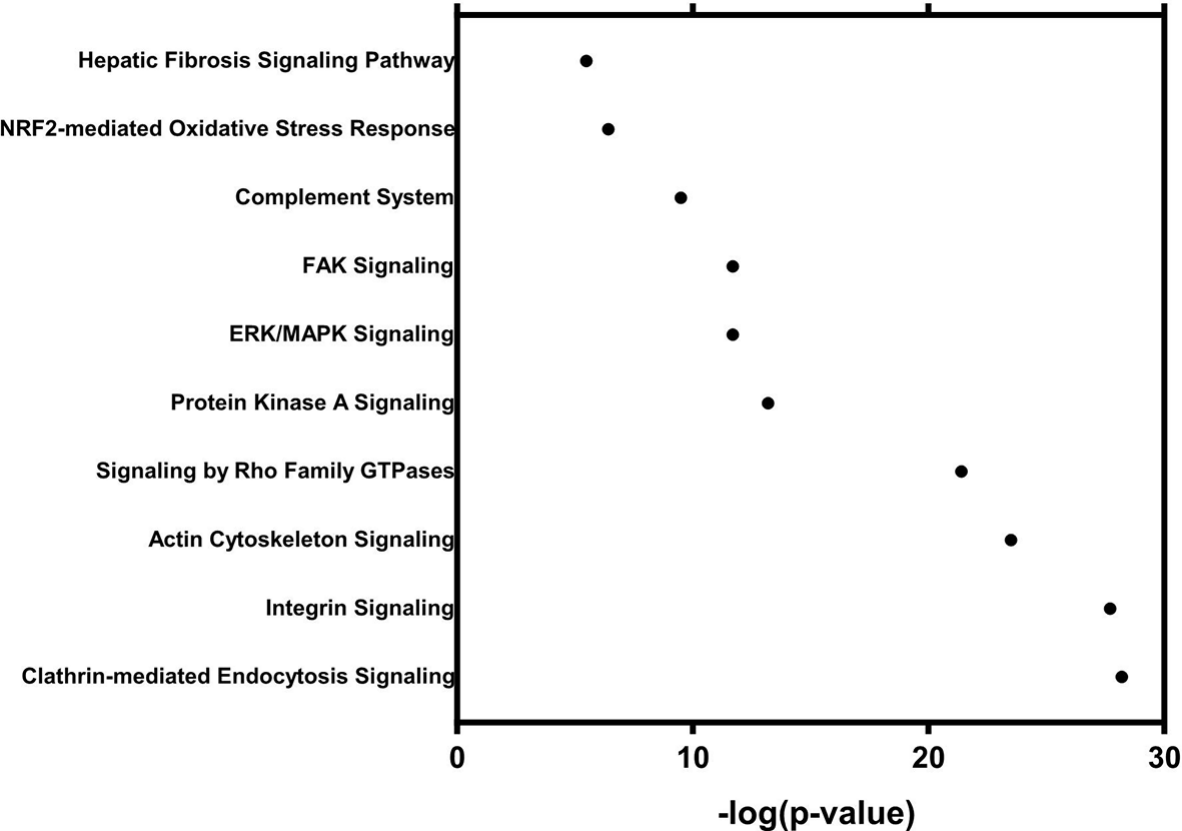
Functional analysis with 1054 differentially expressed proteins in 98 patients.

The mechanistic properties and functions of sEVs in the serum from patients with colorectal neoplasia have not been widely investigated. To further investigate the oncogenic effects of sEV proteins, we performed a functional analysis of the sEV proteome, which included a total of 1921 sEV proteins that showed a statistically significant 2-fold expression difference between the normal and tumor groups. Using IPA for canonical pathway analysis, we conducted functional enrichment analysis of these differentially expressed proteins. The results showed that these differentially expressed proteins were enriched in clathrin-mediated signaling [-log10(p-value) = 28.2], integrin signaling [-log10(p-value) = 27.7], signaling by Rho family GTPases [-log10(p-value) = 21.4], ERK/MAPK signaling [-log10(p-value) = 11.7], and FAK signaling [-log10(p-value) = 11.7] (**Figure 5**). The cellular functions of the six proteins identified by IPA are shown in **Table 2**. The cellular functions and major pathways associated with these six proteins were the clathrin-mediated endocytosis signaling pathway and the complement system in CRC carcinogenesis.

**Table 2:** The cellular function of the six EV proteins identified by IPA.

Gene	Protein	Ingenuity canonical pathway	-Log10 (p-value)
APOF	Apolipoprotein F	Clathrin-mediated endocytosis signaling	28.2
KEL	Kell blood group glycoprotein	Actin cytoskeleton signaling	23.5
PDE5A	cGMP-specific 3’,5’-cyclic phosphodiesterase	Protein kinase A signaling	13.2
CFB	Complement factor B	Complement system	9.49
GCLM	Glutamate-cysteine ligase regulatory subunit	NRF2-mediated oxidative stress response	6.42
ATIC	Bifunctional purine biosynthesis protein ATIC	Hepatic fibrosis signaling pathway	5.48

## Discussion

The current study identified six differentially expressed sEV-associated proteins in the serum of patients with or without colorectal neoplasia, which may serve as biomarkers to diagnose these patients as early as possible. Moreover, several specific signaling pathways intimately related to the progression and development of CRC malignancies were consistently regulated by sEV-associated proteins. Conclusively, we explored the proteomic landscape of sEVs in the serum from patients with colorectal neoplasia with the goal of developing a novel blood test for early diagnosis and shedding new insight into the role of sEVs in CRC progression.

A number of previous studies have evaluated the presence of protein biomarkers in serum for detecting cancer by liquid chromatography coupled to tandem mass spectrometry (23–26). This high-throughput proteomic technique has become a powerful approach for biomarker discovery because it enables the identification and quantification of thousands of proteins within the same experiment. To minimize interference from the high abundance of common proteins in the blood, we focused our attention on serum sEVs, which may contain proteins specific to CRC, to identify meaningful biomarkers of CRC. The present study identified six sEV proteins, namely, GCLM, KEL, APOF, CFB, PDE5A, and ATIC, that could distinguish early neoplasia, advanced neoplasia, and normal controls from each other well. Thus, these six serum sEV proteins are capable of serving as components of a novel blood test to detect precancerous lesions (AAs) and CRC. In contrast, FIT detected CRC with a sensitivity of 79% (27), but the sensitivity decreased to 21–28% for detecting AA (7–9, 28). Moreover, methylated SEPT9 DNA (mSEPT9) is a plasma test approved by the Food and Drug Administration of the United States for detecting CRC. However, its sensitivity for detecting CRC and AA was only 48.2% and 11.2%, respectively (29). Given the suboptimal sensitivity for both CRC and precancerous lesions, the current guidelines, including the US Preventive Services Task Force and American College of Gastroenterology, suggest against the use of mSEPT9 for CRC screening (30, 31). Both FIT and mSEPT9 have limitations in detecting precancerous neoplasms. On the other hand, sEV proteins may distinguish early neoplasia, including AA and stage-I CRC, from normal control cells, thereby providing an opportunity for the development of a new blood test to resolve unmet clinical needs.

The present study identified six sEV proteins differentially expressed between patients with and without colorectal neoplasia and could distinguish AA and CRC from normal controls. The six sEV proteins included GCLM, APOF, ATIC, PDE5A, KEL, and CFB. Understanding the function of these proteins may help to clarify the role of EV in CRC carcinogenesis. GCLM is associated with glutathione biosynthesis and CRC formation. Because GCLM is a sensor of oxidative stress, this association is consistent with the notion that oxidative stress is related to CRC progression (32). APOF is a subtype of apolipoprotein and an essential regulator of cholesterol transport. APO is not only beneficial for diagnosis and prognosis prediction but also a potential therapeutic target in cancer. APO can be abnormally expressed in various cancers, including CRC. In combination with other molecules, APO is a possible biomarker for cancer detection (33). ATIC is a bifunctional enzyme that catalyzes the last two steps of purine biosynthesis. According to a report from The Human Protein Atlas, ATIC has a strong immunoreactivity in CRC tissue (https://www.proteinatlas.org/ENSG00000138363-ATIC). Moreover, ATIC is one of the differentially expressed proteins associated with drug response in CRC (34). PDE5A plays an essential role in signal transduction by regulating the intracellular concentration of cyclic nucleotides (35). Mutation of the PDE5A gene confers a survival advantage in patients with CRC (36). KEL is a zinc endopeptidase with endothelin-3-converting enzyme activity (37). A higher expression of KEL in rectal cancer is associated with worse survival (The Human Protein Atlas, https://www.proteinatlas.org/ENSG00000197993-KEL/pathology/colorectal+cancer/READ). CFB, complement factor B, is a component in the alternative and lectin pathway of the complement system. CFB affects the control of tumor growth, and the activation of the lectin pathway was increased significantly in patients with CRC compared to healthy subjects (38). These abovementioned proteins are encapsulated within sEVs and play a role in CRC carcinogenesis. It would be worthwhile to investigate their role as therapeutic targets in the future.

sEVs play an essential role in cell-cell communication locally and systemically. Growing evidence suggests that the reciprocal relationship between cancer cells and the surrounding nonneoplastic cells, called the tumor microenvironment, is critical for tumor development (39). Thus, great attention has been given to molecules that mediate the cross-talk between cancer cells and the surrounding microenvironment. The role of sEVs in the intercellular exchange of proteins within the cancer microenvironment may play a pivotal role in cancer development, progression, metastasis, and drug resistance in CRC (40, 41). Given the nature of sEVs in intercellular communication, the proteins carried by sEVs have biological functions and actively involved in carcinogenesis. The present study explored whether sEV proteins are involved in CRC carcinogenesis *via* clathrin-mediated endocytosis signaling, the complement system, integrin signaling, ERK/MAPK signaling, FAK signaling, and the complement system. The first two signaling pathways are significantly associated with two of the six EV proteins specific to colorectal neoplasia: APOF and CFB. The other three pathways have been proven to play a critical role in CRC carcinogenesis and progression (42–44).

The functions of the differentially expressed sEV proteins were enriched in the clathrin-mediated endocytosis signaling pathway. The sEV protein specific to colorectal neoplasia, APOF, indeed plays an important role in this related signaling pathway. The clathrin-mediated pathway is the primary pathway for internalizing nutrients, hormones, and other signaling molecules from the plasma membrane into the intracellular compartment and is partially responsible for the cellular immune response and pathogen-influenced signaling. In addition, MACC1 promoted cell proliferation and metastasis *via* a clathrin-mediated pathway in CRC (45). Taken together, clathrin-mediated signaling is associated with cellular maintenance and infectious disease. Consistently, the present study demonstrated that this pathway might play a role in CRC carcinogenesis through sEV proteins.

The other signaling pathway associated with the sEV proteins specific to colorectal neoplasia, namely, CFB, is the complement system. The complement system has traditionally been considered to be related to innate immune responses. Complement components can regulate the tumor microenvironment (TME), functioning as a bridge between tumor-promoting and tumor-suppressing immune responses. Indeed, the balance of the complement components is lost in the presence of cancer (46, 47). In other words, the expression of complement proteins increases in cancer cells, and complement activation in the TME may promote tumorigenesis and progression (48). The mechanism involved in complement activation in the TME remains unclear. The present study explored whether C1q^+^ sEVs increased significantly in subjects with colorectal neoplasia compared with those without. Complement C1q is the activator of the classical pathway. C1q is expressed in several human malignant tumor stroma and vascular endothelium and has been proven to promote cancer cell adhesion, migration, and proliferation (49). The complement proteins could interact with stromal cells in the TME. Tumor-associated macrophages (TAMs) have been reported to contribute to tumor progression (50). The complement proteins could activate and recruit macrophages into tumor tissues. C1q could induce macrophage polarization and suppress macrophage NLRP3 inflammasome activation (51). Complement proteins and TAMs mutually regulate each other. Moreover, recent studies showed that C1q-polarized macrophages expressed elevated levels of programmed death-ligand 1 (PD-L1) and PD-L2 and then suppressed the proliferation of human allogeneic inflammatory T cells (52). Given that PD-L1 is a target molecule for immunotherapy (53), it will be intriguing to investigate the implications of C1q^+^ sEVs in immunotherapy.

Integrin signaling, ERK/MAPK signaling, and FAK signaling are well known to be associated with carcinogenesis. Among these three pathways, integrins work as upstream signaling molecules of the other two pathways. Integrins belong to heterodimeric cell surface adhesion receptors, which are composed of noncovalently associated α and β subunits, and the integrin family consists of 18α and 8β members (54). Each integrin has multiple activation states and exerts effects *via* a cascade of downstream amplification pathways (55). Growing evidence reveals that integrins enable signaling through the cell membrane bidirectionally in addition to physically linking the intracellular cytoskeleton and extracellular matrices (56), thereby controlling cell attachment, movement, growth, differentiation, and survival (57). As cell adhesion receptors, integrins have been reported to be associated with various cancers, including CRC (42). In the present study, we found that the expression of the integrin subunits ITGAM and ITGB2 was increased in the sEVs of patients with colorectal neoplasia. Gong et al. demonstrated that ITGAM was highly expressed in CRC lesions, indicating that the expression of ITGAM is suitable for the diagnosis and prognosis of CRC (42). In addition, Benedicto et al. found that reduced ITGB2 expression was associated with reduced proliferation, adhesion, and migration in various CRC cell lines. This finding suggested a vital role of ITGB2 during CRC metastasis (58). In agreement with the above observations, sEVs, which contain ITGAM and ITGB2, were increased in the serum of patients with colorectal neoplasia, implying that sEVs play a specific role in the expression of these two proteins that participate in the development of cancer malignancies, such as metastasis. Moreover, it is worth investigating whether ITGB2^+^ EVs in serum can be useful for the diagnosis of metastatic CRC.

The ERK/MARK signaling pathway is one of the most important pathways for cell proliferation (59). This pathway is located downstream of many growth factor receptors, including epidermal growth factor and integrins. Activation of this signaling pathway is essential in intestinal epithelial differentiation (60). There is growing evidence that indicates that the activation of the ERK/MAPK pathway is involved in the pathogenesis, progression, and oncogenic characteristics of CRC (43). Our functional proteomic analysis indicated that the expression of sEVs containing the PP1 subunits PPP1CA and PPP1R12A was decreased in subjects with colorectal neoplasia. The decrease in PP1^+^ EVs may minimize their inhibitory effect on ERK/MARK signaling and subsequently promote CRC progression. This hypothesis is supported by a previous study, which demonstrated that lower expression of PPP1R12A was associated with worse recurrence-free survival in CRC (61). FAK, a key mediator of integrin and growth factor signal transduction, is activated directly by SRC kinase (62). The kinase complex of FAK-SRC is reportedly attributed to the activation of downstream pathways, such as PI3K, AKT, and ERK/MARK (63, 64). For example, FAK signaling through the ERK/MARK pathway is needed to promote cancer cell development (65). Moreover, the FAK-SRC complex might induce MMP2 and MMP9 and subsequently increase the invasiveness of cancer cells (66). The present study demonstrated that the expression of the FAK-SRC complex was increased in serum sEVs in patients with colorectal neoplasia. In summary, we investigated whether the differentially expressed sEV proteins may promote the proliferation and metastasis of CRC through upstream integrin signaling, followed by FAK signaling and ERK/MARK signaling. These differentially expressed sEV proteins in patients with colorectal neoplasia will be potential therapeutic targets and worth further investigation in the future.

The strength of the present study is that we explored the proteomic landscape of sEVs in the serum of patients with colorectal neoplasia. The exploration of the proteome in CRC tissue has been conducted in a previous study (67). However, proteomic investigations in serum have been less well-studied in CRC. The advantage of the blood-based approach is that it is more feasible to use the specific sEV proteins as biomarkers for the establishment of a diagnostic test than those from tissue samples. Moreover, six sEV proteins specific to colorectal neoplasia were identified, and these molecules prominently distinguished colorectal neoplasia from the normal control. Our results provide an opportunity to develop a novel blood test for detecting CRC. However, the diagnostic performance, including the sensitivity, specificity, and accuracy of the specific sEV proteins, needs to be validated in the next stage. Thus, one of the limitations of the present study is the lack of validation experiments to support the diagnostic performance of the blood panel test containing the six sEV proteins. Furthermore, the true or false positive rate of the blood panel compared with FIT should be investigated. Another limitation of the present study is that IPA was used to identify the signaling pathways. The biological function of the differentially expressed sEV proteins in the signaling pathways needs to be further validated in the future.

In conclusion, the present study explored whether patients with colorectal neoplasia had distinct expression patterns in the sEV proteome from the serum of patients with colorectal neoplasia compared with those without. Furthermore, six of the differentially expressed sEV proteins were shown to behave as biomarkers to distinguish early neoplasia, advanced neoplasia, and normal controls from each other. These specific sEV proteins have the potential to be used in the development of a novel blood test for detecting colorectal neoplasia at early stages. Finally, the association of the sEV proteome with colorectal neoplasia in CRC carcinogenesis *via* specific signaling pathways was explored. These findings will help improve our understanding of the role of sEV proteins in CRC development and identify candidate therapeutic targets in the future.

## Data Availability Statement

The original contributions presented in the study are included in the article/**Supplementary Material**. Further inquiries can be directed to the corresponding authors.

## Ethics Statement

The studies involving human participants were reviewed and approved by National Taiwan University Hospital, No. 201906020RINC. The patients/participants provided their written informed consent to participate in this study.

## Author Contributions

Conceptualization: T-LS, M-SW, and H-MC. Data curation: KU and H-MC. Formal analysis: Y-CH and C-HK. Funding acquisition: H-MC, T-LS, and L-CC. Investigation: T-LS, KU, and L-CC. Methodology: T-LS, Y-CH, C-HK, and KU. Resources: T-LS, H-MC, and M-SW. Supervision: T-LS. Writing: LC, TS, Y-CH, and C-HK. All authors contributed to the article and approved the submitted version.

## Funding

This work was supported by grants from the Ministry of Science and Technology, Taiwan (MOST109-2314-B-002-085 to L-CC; 110-2321-B-002-014 to M-SW; 105-2320-B-002-058-MY3 to T-LS).

## Conflict of Interest

The authors declare that the research was conducted in the absence of any commercial or financial relationships that could be construed as a potential conflict of interest.

## Publisher’s Note

All claims expressed in this article are solely those of the authors and do not necessarily represent those of their affiliated organizations, or those of the publisher, the editors and the reviewers. Any product that may be evaluated in this article, or claim that may be made by its manufacturer, is not guaranteed or endorsed by the publisher.

## Abbreviations

CRC, colorectal cancer; FIT, fecal immunochemical test; EV, extracellular vesicle; AA, advanced adenoma; LC-MS, liquid chromatography-tandem mass spectrometry; AGC, automatic gain control; GAP, generalized association plot; MV, Matrix visualization; IPA, Ingenuity Pathway Analysis; mSEPT9, methylated SEPT9 DNA; TME, tumor microenvironment; TAM, tumor-associated macrophage.
