# Supplementary material for: Exploration of the Proteomic Landscape of Small Extracellular Vesicles in Serum as Biomarkers for Early Detection of Colorectal Neoplasia
Source: Front Oncol. 2021 Sep 13;11:732743. doi: 10.3389/fonc.2021.732743 (PMC8473825; doi:10.3389/fonc.2021.732743)
Supplement: Supplementary file 1 [file DataSheet_1.docx]

**SUPPLEMENTARY FILE**

This supplementary file has been provided by the authors to give readers additional information about their work.

Supplement to: Chang *et al*. “***Exploration of the proteomic landscape of small extracellular vesicles in serum as biomarkers for early detection of colorectal neoplsia***”.

**Supplementary Figure 1. The expression pattern of proteome between subjects with normal mucosa, early neoplasia, and advanced neoplasia.**

**Figure legend**

Three Matrix visualization maps sorted by HCT–R2E dendrograms for 1921 protein expression on 98 patient’s data. The Euclidean distance map for 98 patients and the Pearson correlation map for 1921 proteins.
